# Suspected Anaphylactic Reactions Associated with Anaesthesia

**DOI:** 10.1111/j.1365-2044.2008.05733.x

**Published:** 2009-02

**Authors:** N J N Harper, T Dixon, P Dugué, D M Edgar, A Fay, H C Gooi, R Herriot, P Hopkins, J M Hunter, R Mirakian, R S H Pumphrey, S L Seneviratne, A F Walls, P Williams, J A Wildsmith, P Wood, A S Nasser, R K Powell, R Mirakhur, J Soar

**Affiliations:** 1British Society for Allergy and Clinical Immunology; 2Royal College of Anaesthetists; 3Resuscitation Council UK

## Summary

The AAGBI has published guidance on management of anaphylaxis during anaesthesia in 1990, 1995 and 2003. This 2008 update was necessary to disseminate new information.Death or permanent disability from anaphylaxis in anaesthesia may be avoidable if the reaction is recognised early and managed optimally.Recognition of anaphylaxis during anaesthesia is usually delayed because key features such as hypotension and bronchospasm more commonly have a different cause.Initial management of anaphylaxis should follow the ABC approach. Adrenaline (epinephrine) is the most effective drug in anaphylaxis and should be given as early as possible.If anaphylaxis is suspected during anaesthesia, it is the anaesthetist’s responsibility to ensure the patient is referred for investigation.Serum mast cell tryptase levels may help the retrospective diagnosis of anaphylaxis: appropriate blood samples should be sent for analysis.Specialist (allergist) knowledge is needed to interpret investigations for anaesthetic anaphylaxis, including sensitivity and specificity of each test used. Specialist (anaesthetist) knowledge is needed to recognise possible non-allergic causes for the ‘reaction’. Optimal investigation of suspected reactions is therefore more likely with the collaboration of both specialties.Details of specialist centres for the investigation of suspected anaphylaxis during anaesthesia may be found on the AAGBI website http://www.aagbi.org.Cases of anaphylaxis occurring during anaesthesia should be reported to the Medicines Control Agency and the AAGBI National Anaesthetic Anaphylaxis Database. Reports are more valuable if the diagnosis is recorded following specialist investigation of the reaction.This guidance recommends that all Departments of Anaesthesia should identify a Consultant Anaesthetist who is Clinical Lead for anaesthetic anaphylaxis.

## Introduction

The AAGBI published its first guidelines on suspected anaphylactic reactions in 1990. Subsequent revisions were published in 1995 and 2003. The current guidelines incorporate advice from a large number of clinical immunologists, allergists and anaesthetists throughout the UK. In common with the 1995 and the 2003 editions, this report is published jointly with the British Society for Allergy and Clinical Immunology (BSACI).

This document is intended to be concordant with, and complementary to, the 2007 Scandinavian Clinical Practice Guidelines, the 2008 Resuscitation Council UK guidelines and the BSACI guidelines: Investigation of suspected anaphylaxis during anaesthesia.

These guidelines apply only to suspected anaphylactic reactions associated with anaesthesia and it is presupposed that the patient is in the care of a trained anaesthetist.

## Objectives

To clarify the definitions used in allergy and anaphylaxis.To review the epidemiology of anaesthesia-related anaphylaxis.To provide advice on the recognition of anaesthetic anaphylaxis.To make recommendations on the immediate management and initial investigation of suspected anaesthetic anaphylaxis.To make recommendations concerning the further investigation of suspected anaesthetic anaphylaxis.To assist anaesthetists in obtaining access to a specialist centre for comprehensive investigation of suspected anaesthetic anaphylaxis.To assist anaesthetists to provide appropriate information to the specialist centre.To make recommendations about the reporting and collection of data on anaesthetic anaphylaxis in Great Britain and Ireland.

## Definitions

The term ‘anaphylaxis’ has been used for all types of acute life-threatening illness triggered by abnormal sensitivity (hypersensitivity) to a trigger agent, and for apparently spontaneous attacks with similar features (idiopathic anaphylaxis). This has made it difficult to define. The EAACI Nomenclature Committee proposed the following broad definition [1]:

Anaphylaxis is a severe, life-threatening, generalized or systemic hypersensitivity reaction.

Minor, localised or non-systemic reactions are outside the definition of anaphylaxis. Anaphylaxis may be divided into ‘allergic anaphylaxis’ and ‘non-allergic anaphylaxis’. The clinical features of allergic anaphylaxis and non-allergic anaphylaxis may be identical. The EAACI committee proposed the term ‘allergic anaphylaxis’ should be used only when the reaction is mediated by an immunological mechanism (such as IgE, IgG, or complement activation by immune complexes). An anaphylactic reaction mediated by IgE antibodies, such as to amoxicillin, is referred to as ‘IgE-mediated allergic anaphylaxis’.

The term ‘anaphylactoid’ reaction had been introduced for non-IgE-mediated anaphylactic reactions but the EAACI committee has recommended this term should no longer be used. This proposal has not been universally accepted. An authoritative recent American practice parameter [2] states: ‘Anaphylaxis is defined … as a condition caused by an IgE-mediated reaction [2]. Anaphylactoid reactions are defined as those reactions that produce the same clinical picture as anaphylaxis but are not IgE mediated.’ In these guidelines we will follow the European (EAACI) nomenclature.

Anaphylaxis is not a homogeneous process: the pathways, mediators, time course and response to treatment depend on the trigger agent, its route and rate of administration, the nature of the patient’s hypersensitivity and the state of health of the patient, including incidental pathology such as respiratory or cardiovascular disease and the effects of concomitant medication such as β-blockers and ACE inhibitors. Although anaphylaxis commonly involves respiratory, cutaneous and circulatory changes, variations such as shock with gastrointestinal disturbance or shock alone are possible. Alternatively, reactions may be fatal without significant shock except as the terminal event following respiratory arrest [3]. Angioedema and urticaria may be features of anaphylaxis but commonly result from mechanisms other than anaphylaxis.

Intravascular volume redistribution is an important component of anaphylactic shock. Cardiac output may be decreased as a result of reduced coronary artery perfusion pressure as well as impaired venous return. Local release of mediators may cause coronary artery spasm and there may be features of acute left or right ventricular failure. Myocardial ischaemia with ECG changes is expected within minutes of anaphylactic shock becoming severe.

Asphyxia may be due to upper airway occlusion caused by angioedema, or bronchospasm with mucus plugging of the lower airways; the latter most commonly occurs in patients taking daily treatment for asthma. Both these processes may occur simultaneously in patients reacting to foods, latex, β-lactam antibiotics or aspirin.

Anaphylaxis usually resolves in 2–8 h but secondary pathology arising from the reaction or its treatment may prolong this. Resolution is complete except when cerebral hypoxia at the peak of the reaction has caused significant brain damage, or when disordered clotting leads to bleeding.

## Acknowledgements

The involvement of the British Society for Allergy and Clinical Immunology and the Clinical Immunology and Allergy section of the British Society for Immunology is gratefully acknowledged. In addition, the working party wishes to acknowledge the assistance of the following:

Sr Alex Farragher Specialist Immunology Nurse

Mr Chris Hirst AAGBI Anaphylaxis website designer

Dr David Noble

Dr Martin Shields

## References

1JohanssonSGOBieberTDahlRRevised nomenclature for allergy for global use: Report of the Nomenclature Review Committee of the World Allergy Organization, October 2003Journal of Allergy and Clinical Immunology200411383261513156310.1016/j.jaci.2003.12.5912Joint Task Force on Practice ParametersThe diagnosis and management of anaphylaxis: an updated practice parameterJournal of Allergy and Clinical Immunology2005115S4835231575392610.1016/j.jaci.2005.01.0103PumphreyRSHLessons for management of anaphylaxis from a study of fatal reactionsClinical and Experimental Allergy2000301144501093112210.1046/j.1365-2222.2000.00864.x

## Epidemiology

### Geographical variation

Most reports on anaesthesia-related anaphylaxis originate from France, Australia, New Zealand and the United Kingdom. Other case series have been described from Scandinavia and the USA. The true incidence and their associated morbidity and mortality remain poorly defined. Both the accuracy and completeness of reporting is not optimal.

10% of anaesthesia-related reactions reported to the UK Medicines Control Agency (MCA) were fatal. These data should be interpreted with caution because it is likely that many less-severe reactions are not reported [1]. Reactions that are accepted as side-effects of certain drugs, for instance, histaminergic reactions due to atracurium and mivacurium, may be under-reported. During a time period of over 6 years only 361 reactions were reported to the MCA. In a 2-year period 789 reactions were reported in France in a comparable population where there is a well-established culture of reporting anaesthesia-related reactions [2].

Based on studies in Australia and France, the incidence of anaphylaxis during anaesthesia has been estimated at between 1 in 10 000 and 20 000 [3, 4] The true incidence of anaphylaxis during anaesthesia in the UK is not known. By extrapolating the French and Australian data to the UK, it is estimated that there are approximately 500 severe reactions in the UK each year. Anaphylactic reactions are more common when drugs are given intravenously.

In one large study an immune basis was demonstrated in two-thirds of patients investigated for anaphylaxis [2]: the remainder comprised non-allergic anaphylaxis and mechanisms other than anaphylaxis. The tests currently available for the diagnosis of anaesthetic anaphylaxis are imperfect. Skin tests and blood tests have limited sensitivity and specificity and their positive and negative predictive value varies between drugs.

### Patient characteristics (age, sex, ethnicity, smoking etc)

Neuromuscular blocking drugs and latex appear to cause anaphylaxis more commonly in female patients. There appears to be a connection between smoking and antibiotic anaphylaxis, possibly because smokers may become sensitised by exposure to repeated courses of antibiotics for respiratory tract infections. Individuals with a history of atopy, asthma or allergy to some foods appear to be at increased risk of latex allergy but not anaphylaxis to neuromuscular blocking drugs or antibiotics [2, 5]. Anaphylaxis associated with radiographic contrast media appears to be associated with atopy [6]. Patients with asthma or taking β-blocking drugs may suffer a more severe reaction. Some of these reactions may be refractory to conventional therapy.

### Possible environmental sensitising agents

The prevalence of anaphylactic reactions to neuromuscular blocking agents (NMBAs) is reported to be at least six times more frequent in some countries [7, 8]: it is considerably more common in Norway than in Sweden. Quaternary ammonium ions (QAI) are proposed to be the allergenic epitopes in NMBAs. Common environmental chemicals such as toothpastes, washing detergents, shampoos, and cough medicines share these allergenic epitopes with the NMBAs [8]. Numerous possibilities exist for a predisposed individual to become sensitised to QAIs and thus be at risk of developing anaphylaxis to NMBAs during anaesthesia. In a recent Scandinavian survey, use of certain cough medicines was found to be the only significant difference in environmental chemical exposure [8]. In Norway, but not Sweden, cough syrups containing pholcodine are available without prescription. IgE-antibodies to pholcodine were seen in 6% of a general population (blood donors) in Norway but not in Sweden (0%).

### Muscle relaxants

Approximately 60% of cases of anaesthesia-related anaphylaxis are thought on the basis of skin tests to be due to neuromuscular blocking agents. Mivacurium and atracurium are associated with non-allergic anaphylaxis in which release of mediators (notably histamine) from mast cells exactly imitates allergic anaphylaxis. Cisatracurium, although sharing a benzylisoquinolinium structure, is not associated with non-allergic anaphylaxis, although several cases of allergic anaphylaxis have been reported. It is generally accepted that succinylcholine is the NMBA most likely to be associated with allergic anaphylaxis, although rocuronium has been implicated in a similar number of cases in France.

The prevalence of sensitisation to NMBAs in the community is higher than the incidence of reactions would suggest. In one study, approaching 10% of the general population exhibited skin reactivity to NMBAs, an incidence that far exceeds the incidence of anaphylaxis on administration of these drugs during anaesthesia [9]. A previous history of specific drug exposure is not necessary, particularly for neuromuscular blocking drugs. A history of previous exposure is found in fewer than 50% of patients who are allergic to neuromuscular blocking drugs. Conversely, an uneventful exposure may sensitise an individual to subsequent administration of the drug.

Cross-sensitivity between different NMBAs is relatively common, probably because they share a quaternary ammonium epitope. If anaphylaxis to an NMBA is suspected, the patient should undergo skin prick testing with all the NMBAs in current use. If a patient demonstrates a positive skin prick test (SPT) to an NMBA, the patient should be warned against future exposure to all NMBAs if possible. If it is mandatory to use an NMBA during anaesthesia in the future, it would seem appropriate to permit the use of an NMBA which has a negative skin test, accepting that a negative skin test does not guarantee that anaphylaxis will not occur.

The apparent excess of cases of anaphylaxis to rocuronium in some countries should be interpreted with caution until much more data have been collected. Large sample sizes are needed to estimate the true incidence: if the true incidence is 1 in 5000, a sample size of 7 million would be needed to have a 95% chance of being within 5% of the true value [10]. There is a need for further epidemiological studies.

### Latex

Latex hypersensitivity is the second most common cause of anaesthesia-related anaphylaxis in many studies (up to 20% of cases). However, the incidence of anaphylaxis to latex is considerably less than would be suggested by the prevalence of positive skin tests or positive IgE tests. It appears that the incidence may be waning, at least in some countries; possibly as a result of a change or decline in the use of latex gloves. Certain patient groups are more susceptible to latex anaphylaxis (see Appendix II).

### Antibiotics

Approximately 15% of anaesthesia-related anaphylactic episodes are due to antibiotics. This proportion has increased in recent years and may merely mirror increased exposure to antibiotics in the community [2, 11]. The pre-operative history is important. Although only a minority of patients who report allergy to antibiotics have a true allergy, the consequence of anaphylaxis to intravenous antibiotics may be catastrophic, and self-reporting should be taken seriously. Skin testing is only approximately 60% predictive of clinical hypersensitivity. Penicillins and cephalosporins which share the β-lactam ring are responsible for approximately 70% of antibiotic-induced anaphylaxis. Many antibiotics possess a β-lactam ring:

Benzylpenicillin, phenoxymethylpenicillinFlucloxacillin, temocillinAmoxicillin, ampicillin, co-amoxiclavCo-fluampicilPiperacillin, ticarcillin,Pivmecillinam HClCephalosporinsAztreonamErtapenem, imipenem with cilastatin, meropenem

The structure of the side chains attached to the β-lactam ring is also important in determining the response of the immune system. First generation cephalosporins and cefamandole share a similar side chain with penicillin and amoxicillin. A recent meta-analysis suggested that patients who are allergic to penicillin or amoxicillin have a higher incidence of allergic reactions to first generation cephalosporins and cefamandole, but not other cephalosporins [12]. The issue is complicated because the classification of cephalosporins relates to their antimicrobial activity rather than their chemical structure.

### First generation cephalosporins

Cefalexin (cephalexin)*CephaloridineCephalothinCefazolinCefradine (cephradine)*Cefadroxil *

### Second generation cephalosporins

Cefaclor*CefamandoleCefuroxime*

### Third generation cephalosporins

Cefixime*Cefotaxime*Cefpodoxime*Ceftazidime*Ceftriaxone*

### Fourth generation cephalosporins

CefepimeCefpirome

*Listed in the British National Formulary.

### Local anaesthetics

Anaphylactic reactions to local anaesthetic drugs are very uncommon. Local anaesthetic esters are more likely than amides to provoke a Type lV allergic reaction. Preservatives such as methyl-paraben or metabisulphites may be responsible in some cases. It has been suggested that inadvertent intravascular injection of a local anaesthetic or the systemic absorption of adrenaline may be responsible for many of the reported reactions. Reactions occurring in the dental chair may also be associated with idiopathic angioedema or latex allergy.

### Opioids

Opioids are an uncommon cause of anaesthesia-related anaphylaxis. Diagnosis is difficult and the true incidence is unknown. Morphine, pethidine and codeine are well-known to cause non-specific histamine release which precludes diagnostic skin testing. The diagnosis of opioid anaphylaxis often rests on a careful history and the exclusion of other possibilities. Challenge testing may be appropriate in some cases but may be performed only in specialist centres.

### Anaesthetic induction agents

Anaphylaxis to propofol is very uncommon. The antigenic determinant may be the isopropyl groups. It has been stated that patients with egg allergy or soy allergy should avoid propofol but there is no strongly-supportive evidence. It has been suggested that propofol anaphylaxis is more likely if lignocaine is added to reduce pain on injection: there is no evidence to support this suggestion. Anaphylaxis to thiopental has become extremely uncommon, probably reflecting the decline in its use. A small number of cases of midazolam anaphylaxis have been reported.

### Non-steroidal anti-inflammatory drugs (NSAIDs)

Several mechanisms may be responsible for reactions to NSAIDs. Inhibition of the PGE_2_ pathway leads to excessive leukotriene synthesis and subsequent mediator release, causing urticaria or bronchospasm. IgE-mediated reactions may also occur in relation to some NSAIDs. Fatal anaphylaxis has been described after oral administration of NSAIDs.

### Halogenated volatile anaesthetics

There are no published reports of anaphylaxis to halogenated volatile anaesthetics. The rare fulminant form of hepatitis associated with halothane has an immune basis which is unrelated to anaphylaxis.

### Colloids

Intravenous colloids are responsible for approximately 4% of all peri-operative anaphylactic reactions. In one study, gelatin solutions were responsible for 95% of the intravenous colloid reactions. It has been stated that the incidence may be greater with the urea-linked gelatins compared with the modified fluid gelatins. Intravenous gelatin solutions should be avoided in patients with a history of allergy to gelatin-containing vaccines. Anaphylaxis to intravenous dextrans has been reported, with an incidence similar to the modified fluid gelatins. Anaphylaxis to hydroxyethyl-starch is rare.

### Antiseptics and disinfectants

Reactions to chlorhexidine have come into greater prominence in recent years. There appears to be a significant difference in incidence between countries [13]. Reactions range from contact dermatitis to life-threatening anaphylaxis. Anaphylaxis has occurred when chlorhexidine was used as an antiseptic for urological and gynaecological procedures as well as insertion of central venous and epidural catheters. The chlorhexidine coating of certain central venous catheters has been implicated in such reactions. It is prudent to allow skin disinfectant to completely dry before beginning an invasive procedure. Anaphylaxis to polyvinylpyrrholidine as povidone-iodine or as an excipient for oral medicines occurs but is rare.

### Miscellaneous agents

Many agents to which patients may be exposed during anaesthesia may be associated with anaphylaxis, including aprotinin, protamine, heparins, radiological contrast material, dyes and oxytocin. Anaphylaxis to glycopyrronium and neostigmine may occur very rarely.

## References

1AxonADHunterJMEditorial III: Anaphylaxis and anaesthesia – all clear now?British Journal of Anaesthesia20049350141536147510.1093/bja/aeh2032MertesPMLaxenaireMCAllaFAnaphylactic anaphylactoid reactions occurring during anaesthesia in France in 1999–2000Anesthesiology200399536451296053610.1097/00000542-200309000-000073FisherMMBaldoBAThe incidence and clinical features of anaphylactic reactions during anesthesia in AustraliaAnnales Francaises d’Anesthesie et de Reanimation19931297104836859210.1016/s0750-7658(05)81016-04LaxenaireMCEpidemiology of anesthetic anaphylactoid reactions. Fourth multicenter survey (July 1994-December 1996)Annales Francaises d’Anesthesie et de Reanimation1999187968091048663410.1016/s0750-7658(00)88460-95LaxenaireMCMertesPMAnaphylaxis during anaesthesia. Results of a two-year survey in FranceBritish Journal of Anaesthesia200187549581187872310.1093/bja/87.4.5496LangDMAlpernMBVisintainerPFSmithSTIncreased risk for anaphylactoid reaction from contrast media in patients on beta-adrenergic blockers or with asthmaAnnals of Internal Medicine19911152706167723910.7326/0003-4819-115-4-2707LaakeJHRottingenJARocuronium and anaphylaxis – a statistical challengeActa Anaesthesiologica Scandinavica20014511962031173666910.1034/j.1399-6576.2001.451004.x8FlorvaagEJohanssonSGOmanHPrevalence of IgE antibodies to morphine. Relation to the high and low incidences of NMBA anaphylaxis in Norway and Sweden, respectivelyActa Anaesthesiologica Scandinavica200549437441577728910.1111/j.1399-6576.2004.00591.x9PorriFLemiereCBirnbaumJPrevalence of muscle relaxant sensitivity in a general population: implications for a preoperative screeningClinical and Experimental Allergy1999297251005170410.1046/j.1365-2222.1999.00453.x10FisherMBaldoBAAnaphylaxis during anaesthesia: current aspects of diagnosis and preventionEuropean Journal of Anaesthesiology19941126384792533311MossJAllergy to anestheticsAnesthesiology20039952131296053310.1097/00000542-200309000-0000312PichicheroMECaseyJRSafe use of selected cephalosporins in penicillin-allergic patients: a meta-analysisOtolaryngology-Head and Neck Surgery200713634071732185710.1016/j.otohns.2006.10.00713GarveyLHRoed-PetersenJMenneTHusumBDanish Anaesthesia Allergy Centre – preliminary resultsActa Anaesthesiologica Scandinavica200145120491173667010.1034/j.1399-6576.2001.451005.x

## Recognition and clinical diagnosis of anaphylaxis

In a closely monitored patient such as during anaesthesia, when a fall in blood pressure, change in heart rate or difficulty with ventilation are noticed, anaphylaxis is so rarely the cause that inevitably treatment is given for another diagnosis before anaphylaxis is recognised; the response to this treatment has commonly delayed or even prevented recognition of the true cause. It is therefore probable that many grade I and II acute allergic reactions to anaesthetic drugs are missed. Conversely, a cause other than allergic anaphylaxis seems more likely in around one-third of the patients referred to specialist centres for investigation of suspected anaphylaxis during anaesthesia.

### Previous history

Any clue that appropriately raises anticipation of anaphylaxis will be helpful. A previous history of reaction to anaesthetic drugs, antibiotics, other drugs, chlorhexidine or latex should obviously lead to appropriate avoidance. Because it is always possible the wrong trigger factor was identified, the exact details of exposure leading to any previous reaction should be sought during the pre-operative assessment. Cross-reactivity between non-anaesthetic drugs such as pholcodine or environmental chemicals containing quaternary ammonium groups such as cosmetics and detergents has been proposed as the source of sensitivity to opioids and muscle relaxants: a history of cutaneous sensitivity to cosmetics or rashes from cough medicines should raise caution.

Anaphylaxis to amoxicillin and cephalosporins is commonest in asthmatic smokers who have had multiple courses of these antibiotics without reacting: the symptoms of anaphylaxis in such a patient are likely to be initially misinterpreted (quite reasonably) as those expected from anaesthesia in an asthmatic smoker.

### Clinical features

Although anaesthesia-related anaphylaxis usually results from intravenous drug administration, administration by other routes, for example cutaneous, mucosal/vesical peritoneal, intra-articular or intramuscular may be responsible. Clinical features include hypotension, tachycardia or bradycardia; cutaneous flushing, rash or urticaria; bronchospasm; hypoxia; angioedema and cardiac arrest. If an adverse event such as hypotension or bronchospasm occurs during anaesthesia it is appropriate to suspect anaphylaxis unless there exists a significantly more likely cause.

Tachycardia is not invariable: bradycardia is seen in approximately 10% of patients with allergic anaphylaxis during anaesthesia. Hypotension is the sole clinical feature in approximately 10% of patients and is likely to be exaggerated in patients undergoing anaesthesia with neuraxial blockade. Widespread flushing or urticaria is seen in the majority of patients but the absence of cutaneous signs does not exclude anaphylaxis. Bronchospasm may be more common in patients with pre-existing asthma.

**Table d32e981:** 

Clinical features	Allergic anaphylaxis (*n* = 518), *n* (%)	Non-allergic anaphylaxis (*n* = 271), *n* (%)
Cardiovascular	387 (74.7)	92 (33.9)
Arterial hypotension	90 (17.3)	50 (18.4)
Cardiovascular collapse	264 (50.8)	30 (11.1)
Bradycardia	7 (1.3)	2 (0.7)
Cardiac arrest	31 (5.9)	–
Bronchospasm	207 (39.8)	52 (19.2)
Cutaneous signs	374 (71.9)	254 (93.7)
Angioedema	64 (12.3)	21 (7.7)

Clinical features of allergic anaphylaxis and non-allergic anaphylaxis occurring during anaesthesia in France between 1st Jan 1999 and 31st Dec 2000. Mertes PM *et al.Anesthesiology* 2003; **99**: 536–45.

The clinical features usually occur within a few minutes but may be delayed by up to an hour. Substances to which the clinical reaction may be delayed include latex, antibiotics, intravenous colloids and Cidex OPA (used to disinfect surgical instruments). However, an immediate response does not exonerate these substances. The clinical features of anaphylaxis to neuromuscular blocking agents usually develop rapidly. Deflation of a surgical tourniquet may induce anaphylaxis if the allergen has been sequestered in the limb.

## Immediate management

These management guidelines presuppose that the patient is in the care of an appropriately-trained anaesthetist and full resuscitation facilities and appropriate vital signs monitors are available.

There is a wide spectrum of severity and combinations of clinical features. Although management should be tailored to the individual patient, there is consensus that adrenaline should be given as early as possible. In addition to having alpha-agonist activity, adrenaline is a valuable beta-agonist which is inotropic and a bronchodilator, and reduces further mediator release.

Other causes of hypotension or difficulty in ventilation should be excluded, for example a misplaced tracheal tube or equipment failure.

## Immediate management

Use the ABC approach (Airway, Breathing, Circulation). Team-working enables several tasks to be accomplished simultaneously.Remove all potential causative agents (including IV colloids, latex and chlorhexidine) and maintain anaesthesia, if necessary, with an inhalational agent.Call for help and note the time.Maintain the airway and administer oxygen 100%. Intubate the trachea if necessary and ventilate the lungs with oxygen.Elevate the patient’s legs if there is hypotension.If appropriate, start cardiopulmonary resuscitation immediately according to Advanced Life Support Guidelines.Administer adrenaline intravenously. An initial dose of 50 μg (0.5 ml of 1 : 10 000 solution) is appropriate (adult dose). Several doses may be required if there is severe hypotension or bronchospasm.If several doses of adrenaline are required, consider starting an intravenous infusion of adrenaline (adrenaline has a short half-life).Administer saline 0.9% or lactated Ringer’s solution at a high rate via an intravenous cannula of an appropriate gauge (large volumes may be required).

## Secondary management

Administer chlorphenamine 10 mg IV (adult dose).Administer hydrocortisone 200 mg IV (adult dose).If the blood pressure does not recover despite an adrenaline infusion, consider the administration of an alternative intravenous vasopressor according to the training and experience of the anaesthetist, for example metaraminol.Treat persistent bronchospasm with an intravenous infusion of salbutamol. If a suitable breathing-system connector is available, a metered-dose inhaler may be appropriate. Consider giving intravenous aminophylline or magnesium sulphate.Arrange transfer of the patient to an appropriate Critical Care area.Take blood samples (5–10 ml clotted blood) for Mast Cell Tryptase as follows:Initial sample as soon as feasible after resuscitation has started – do not delay resuscitation to take the sample.Second sample at 1–2 h after the start of symptoms.Third sample either at 24 h or in convalescence (for example in a follow-up allergy clinic). This is a measure of baseline tryptase levels as some individuals have a higher baseline level.Ensure that the samples are labelled with the time and date.Liaise with the hospital laboratory (see Appendix lll: Mast cell Tryptase).

## Drug doses in children

### Adrenaline

#### Intramuscular

> 12 years: 500 μg IM (0.5 ml of a 1 : 1000 solution)

300 μg IM (0.3 ml of a 1 : 1000 solution) if the child is small

6–12 years: 300 μg IM (0.3 ml of a 1 : 1000 solution)

Up to 6 years: 150 μg IM (0.15 ml of a 1 : 1000 solution)

#### Intravenous

Intravenous adrenaline may be used in children in acute areas such as operating theatres or intensive care units by those familiar with its use and if IV access is already available. Great care should be taken to avoid dose errors when preparing drug dilutions. Prepare a syringe containing 1 ml of 1 : 10 000 adrenaline for each 10 kg body weight (0.1 ml.kg^−1^ of 1 : 10 000 adrenaline solution = 10 μg.kg^−1^. Titrate to response, starting with a dose of one-tenth of the contents of the syringe, i.e. 1 μg.kg^−1^. Often a child will respond to as little as 1 μg.kg^−1^. In smaller children, further dilution may be needed to allow dose titration (check carefully for decimal point and concentration errors). The intramuscular route is preferred where there is no venous access or where establishing venous access would cause a delay in drug administration.

### Hydrocortisone

> 12 years: 200 mg IM or IV slowly

6 to 12 years: 100 mg IM or IV slowly

6 months to 6 years: 50 mg IM or IV slowly

< 6 months: 25 mg IM or IV slowly

### Chlorphenamine

> 12 years: 10 mg IM or IV slowly

6 to 12 years: 5 mg IM or IV slowly

6 months to 6 years: 2.5 mg IM or IV slowly

< 6 months: 250 μg.kg^−1^ IM or IV slowly

## Later investigations to identify the causative agent

Any patient who has a suspected anaphylactic reaction associated with anaesthesia should be investigated fully, but investigations should not interfere with the immediate treatment of the patient. The anaesthetist who administered the anaesthetic or the consultant anaesthetist in charge of the patient is responsible for ensuring that the reaction is investigated.

The patient should be referred to a specialist Allergy or Immunology centre that has appropriate experience of investigating this type of problem. *Anaesthetic departments should identify a lead anaesthetist for anaesthetic anaphylaxis. Referral pathways should be agreed prospectively with the appropriate specialist centre.*

## Criteria for referral to a specialist centre for investigation

Patients in whom allergic or non-allergic anaphylaxis is suspected should be referred to a specialist clinic for investigation. A patient should be referred if there is any of the following:

Unexplained cardiac arrest during anaesthesia.Unexplained, unexpected hypotension (for example a decrease of mean arterial pressure of more than 30 mmHg) which requires active treatment.Unexplained, unexpected bronchospasm, particularly if the bronchospasm is severe, causes a significant decrease in oxygen saturation and is relatively resistant to treatment.Widespread rash, flushing or urticaria.Angioedema.

A detailed analysis of events surrounding the suspected anaphylactic reaction must be undertaken. The time of onset of the reaction in relation to induction and other events is the most important information. All drugs and other agents to which the patient was exposed before and during the anaesthetic as well as their timing in relation to the reaction must be recorded.

Details sent to the specialist centre along with a letter of referral must include:

A legible photocopy of the anaesthetic recordA legible photocopy of the recovery room chartLegible photocopies of drug chartsA description of the reaction and time of onset in relation to inductionDetails of blood tests sent and their timing in relation to the reactionContact details of the surgeon and the general practitioner

A standard referral proforma is useful (see Appendix S1 in Supporting Information).

## Tests performed at the specialist centre

### Skin tests

Patients should be referred for skin testing as soon as possible after the clinical event. Skin testing can be performed as soon as the patient has made a full clinical recovery and the effects of any antihistamine given to treat the reaction have fully worn off. Skin tests can provide confirmation of sensitisation to a specific drug but must be interpreted within the clinical context. There are a variety of techniques and several different criteria are used for positivity [1–4] so skin testing must be performed by suitably trained and experienced personnel.

A histamine solution and physiological saline are used as positive and negative controls respectively. Drugs with anti-histamine activity must be discontinued a few days before testing. There is no need to discontinue oral or inhaled steroids.

Skin prick tests are usually done on the volar surface of the forearm. A drop of the drug is placed on the skin and the skin pricked through the drop with a lancet. The results are read after 15–20 min.

Intradermal tests may be performed where skin prick tests are negative. Although they are more sensitive, they are less specific, more difficult to interpret and more likely to precipitate a systemic reaction. Intradermal tests are usually performed on the forearm or back. 0.02 to 0.05 ml of a dilute solution is injected intradermally and the results read after 20–30 min.

Both skin prick tests and intradermal tests are widely used in the diagnosis of IgE mediated allergic reactions though the diagnostic value of a positive test for most drugs is limited due to lack of subsequent challenge data. If the mechanism is unknown a negative test is unreliable.

All drugs used during anaesthesia may be tested and lists of non-irritant dilutions have been compiled for both skin prick tests and intradermal tests [5]. Skin tests are most useful for latex, beta lactam antibiotics and NMBAs. They are also useful for induction agents, protamine [6] and chlorhexidine. Intradermal testing may be more reliable for propofol [2]. Skin tests are not usually performed for opioids because false positive results are common. However, skin prick testing appears to be informative for the synthetic opioids fentanyl and remifentanil. Skin tests are not useful for NSAIDs, dextrans or iodinated radiological contrast media [6] because anaphylaxis to these agents is not usually IgE-mediated.

Patch tests are the mainstay of diagnosis for contact allergic reactions and may be useful for diagnosing exanthematous drug rashes but are not helpful in eliciting the cause of suspected anaphylactic reactions occurring during anaesthesia.

## References

1PepysJPepysEOBaldoBAWhitwamJGAnaphylactic/anaphylactoid reactions to anaesthetic and associated agentsAnaesthesia1994494705801758810.1111/j.1365-2044.1994.tb03515.x2FisherMMcDBoweyCJIntradermal compared with prick testing in the diagnosis of anaesthetic allergyBritish Journal of Anaesthesia1997795963930139010.1093/bja/79.1.593BergCMHeierTWilhelmsenVFlorvaagERocuronium and cisatracurium-positive skin tests in non allergic volunteers: determination of drug concentration thresholds using a dilution titration techniqueActa Anaesthesiologica Scandinavica200347576821269951610.1034/j.1399-6576.2003.00093.x4LeynadierFSansarricqMDidierJMDryJPrick tests in the diagnosis of anaphylaxis to general anaestheticsBritish Journal of Anaesthesia1987596839360691210.1093/bja/59.6.6835Prevention of allergic risk in AnaethesiaSociete Francaise d'Anesthesie et de Reanimation http://www.sfar.org/allergiefr.html [accessed 8 October 2008]6FisherMBaldoBAAnaphylaxis during anaesthesia: current aspects of diagnosis and preventionEuropean Journal of Anaesthesiology199411263847925333

## Blood tests

Appropriate further blood investigations depend on the drug in question, and the experience of the specialised laboratory service. Blood samples for specific IgE tests may be taken at the time of the reaction, or soon afterwards during that hospital admission. If the results are negative these tests should be repeated when the patient is seen at the specialist centre in case the initial results might be falsely negative due to possible consumption of relevant IgE antibodies during the reaction. The most commonly used test for allergen-specific IgE uses target allergen or drug bound onto a three-dimensional sponge-like solid matrix or ‘CAP’ and a fluorescent detection system. The Radio-AllergoSorbent Test (RAST) uses a radioactive detection system: it is now rarely used but the name has persisted in common use.

Specific IgE antibodies against succinylcholine (thiocholine ester) can be assayed in serum but the sensitivity is relatively poor (30–60%). Tests for serum IgE antibodies against other neuromuscular blocking drugs are not available in the UK. Specific IgE antibodies against several antibiotics can be assayed. These include amoxicilloyl, ampicilloyl, penicilloyl G, penicilloyl V and cefaclor, however the diagnostic value of these tests is less well defined. Tests for specific antibodies against latex, chlorhexidine and bovine gelatin are available.

The presence of drug-specific IgE in serum is evidence of allergic sensitisation in that individual and provides a possible explanation of the mechanism and specific drug responsible for the reaction. It is not in itself proof that the drug is responsible for the reaction. It is important to emphasise that attribution of cause and effect is a judgement made after considering all the clinical and laboratory information relevant to the reaction.

## Experimental tests

There is much research into ways of improving the in vitro elucidation of anaphylaxis. In addition to tissue-bound mast cells, circulating basophils are also involved in anaphylaxis. During anaphylaxis, proteins such as CD63 and CD203c become newly or increasingly expressed on the surface of basophils. These can be detected by flow cytometry which forms the basis of experimental drug-induced basophil stimulation tests.

## Appendix I

### Frequently asked questions

#### Should I give a test dose of IV antibiotic?

No. Predictive testing would require serial challenges with increasing doses, starting with a minuscule dose and allowing at least 30 min between each dose. This approach is impossible within the constraints of an operating list.

#### Should I avoid cephalosporins in a patient who gives a history suggestive of penicillin allergy?

Most patients who give a history of a penicillin-related rash are not allergic to cephalosporins. However, there are now many suitable alternatives to cephalosporins and, unless there are compelling bacteriological indications, it would be appropriate to consider avoiding first and second generation cephalosporins. If there is a convincing history of penicillin-related *anaphylaxis*, the case for avoiding first and second generation cephalosporins is stronger.

#### Should I use crystalloid or colloid in the immediate management of anaphylaxis?

There is no evidence that one is better than the other. If an IV colloid has already been given to the patient before the appearance of clinical signs of anaphylaxis, it should be discontinued and either replaced by crystalloid or replaced by a colloid of a different class; e.g. replace a gelatin-based colloid with a starch-based colloid.

#### Should I give an H2-blocking drug as part of the immediate management of anaphylaxis?

There is no evidence to support the use of H2-blocking drugs in this situation.

#### What should I say to a patient who wishes to be screened for anaesthetic allergy pre-operatively?

Unless there is a history suggestive of previous anaesthetic anaphylaxis, pre-operative screening is of no value. The sensitivity and specificity of skin tests and blood tests is relatively low. If the pretest probability is very low, i.e. there is no positive history, neither a negative test nor a positive test is likely to be predictive of outcome.

#### Should I avoid propofol in patients who are allergic to eggs, soya or nuts?

There is no published evidence indicating that propofol should be avoided in patients who are allergic to eggs, soya or nuts. Propofol contains purified egg phosphatide and soya-bean oil. It is likely that the manufacturing process removes or denatures the proteins responsible for egg allergy and soya allergy. However, a cautious approach would seem to be appropriate in patients with egg allergy or soya allergy.

#### I don’t have a specialist allergy centre nearby. Should I do my own skin-testing?

The results of skin tests are very technique-dependent and their interpretation requires specialist training and experience.

### What should I do if a patient with a convincing history of cardio-respiratory collapse during a previous anaesthetic presents for emergency surgery without having been investigated for anaphylaxis?

1. Covert cardiac or respiratory disease should be sought in accordance with normal pre-operative practice.
2. It may be possible to exclude latex allergy by taking a detailed history from the patient. If this is impossible, a latex-free environment should be provided.
3. Inhalational agents are likely to be safe unless the previous collapse was due to malignant hyperthermia.
4. If previous records are available:
5. It would be appropriate to avoid all drugs given during the previous anaesthetic before the onset of cardio-respiratory collapse, with the exception of inhalational agents.
6. If the patient received a neuromuscular blocking drug before the collapse, then all neuromuscular blocking drugs should be avoided if possible because cross-sensitivity is common.
7. If previous records are not available:
8. It would be appropriate to avoid all neuromuscular blocking drugs if possible (the anaesthetist will need to assess the balance of risks).
9. Drugs used in local and regional anaesthesia are likely to be safe; allergy to amide local anaesthetic drugs is extremely rare.
10. It would be appropriate to avoid chlorhexidine preparations if possible; allergy to povidone iodine is less common than allergy to chlorhexidine.
11. The previous reaction may have been the result of non-allergic anaphylaxis; it would be appropriate to avoid drugs that are known to release histamine, for example morphine.
12. There is no evidence that pre-treatment with hydrocortisone or histamine-blocking drugs will reduce the severity of anaphylaxis.

## Appendix II

### Latex allergy

Latex is a natural rubber derived from the sap of a tree (*Hevea brasiliensis*) found in Asia which contains several water-soluble proteins, designated Hev b 1- Hev b 13. Patient contact with natural rubber latex (NRL) during anaesthesia may occur via several routes including the airway, mucous membranes, surgical wounds and parenteral injection. An anaphylactic reaction to NRL during anaesthesia may be immediate, or may be delayed for up to an hour. The clinical manifestations of NRL anaphylaxis during anaesthesia tend to be less severe than anaphylaxis due to neuromuscular blocking drugs.

#### High risk individuals

Approximately 8% of the population is sensitized to latex but only 1.4% of the population exhibits latex allergy. The following groups are at increased risk:

- Patients with atopy
- Children undergoing multiple surgical procedures, such as spina bifida, or children undergoing surgery at a very young age
- Patients with severe dermatitis of their hands
- Healthcare professionals
- Patients with allergy to fruits; most frequently banana, chestnut and avocado
- Industrial workers using protective gear
- Occupational exposure to latex

#### Pre-operative assessment

A history of pre-operative symptoms suggestive of latex allergy is present in approximately a quarter of patients who develop intra-operative NRL anaphylaxis. A thorough history, including an occupational history, should be part of the pre-operative assessment. It is useful to ask specifically whether contact with balloons, condoms or latex gloves provokes itching, rash or angioedema.

#### Pre-operative testing

If the clinical history is positive or equivocal, the patient should be referred for testing before the surgical procedure and surgery should proceed only in an emergency. In the preoperative situation, it is particularly important to exclude IgE-mediated sensitivity. The choice of test: either a blood test for latex-specific IgE, or skin testing may depend on local availability. Current tests have a sensitivity of approximately 75–90%; skin prick testing may be more sensitive than blood tests for specific IgE. It is important that skin prick tests are performed by trained staff. It may be appropriate to proceed to challenge testing in some patients.

#### Peri-operative precautions

- If NRL allergy is diagnosed pre-operatively, avoidance is mandatory.
- Care should be taken to avoid introducing additional, unrelated hazards when latex precautions are implemented in the operating theatre.
- Latex allergy should be recorded in the case-notes and on the patient’s wrist bracelet.
- The surgical team and the nursing and anaesthetic support teams should be alerted.
- There is no evidence that premedication with an anti-histamine or steroid is useful.
- The operating theatre should be prepared the night before to avoid the release of latex particles and the patient should be scheduled first on the list. Evidence is limited regarding the need for this degree of caution in operating theatres where all latex gloves are of the non-powdered type.
- A ‘Latex allergy’ notice should be placed on the doors to the anaesthetic room and operating theatre.
- Staff traffic should be limited.
- Absolute use of synthetic gloves when preparing equipment and during anaesthesia, surgery and postoperative care.
- Non-essential equipment should be removed from the vicinity of the patient.
- The anaesthetic and surgical areas must contain only latex free products.
- Only latex-free dressings and tapes should be applied.
- Equipment in resuscitation boxes and trolleys must contain only latex free material.

#### Postoperative investigation of possible latex anaphylaxis

If skin testing and specific IgE testing is negative or equivocal in the presence of a suggestive history, it may be appropriate for the patient to proceed to a provocation test with the finger of a latex glove. This procedure must be performed by trained staff and resuscitation facilities must be available. Patch testing by an expert is useful in the diagnosis of delayed (Type lV) hypersensitivity to rubber.

#### Hospital Latex-Allergy Policy

It is recommended that all hospitals have a latex-allergy policy which should address the following:

- A named clinical lead for latex allergy
- Instructions for accessing latex-allergy testing
- Latex allergy precautions in the ward and clinic settings
- Latex allergy precautions in the operating theatre environment
- Avoidance of latex sensitization in employees
- Liaison with pharmacy and equipment-procurement departments

### Classification of latex reactions

1. Systemic reaction: a Type 1 hypersensitivity reaction involving specific IgE to Hev proteins, mostly to Hev b 5&6. This is the most severe but least frequent reaction and occurs in genetically predisposed individuals. The symptoms consist of itching, swelling, rhinitis, asthma and anaphylaxis. This allergy is life long. A proportion of sensitized individuals also react to certain fruits cross reacting with latex proteins.
2. Contact dermatitis: a delayed Type IV hypersensitivity reaction consisting of an eczematous reaction starting 24–48 h after repeated skin or mucosal contact with additives used during rubber production. This is a T cell-mediated, non-life-threatening reaction. It may, however, predispose the individual to a more severe systemic reaction.
3. Clinical non-immune-mediated reaction: irritant dermatitis resulting from a mild irritant effect of the powder in the gloves and that of disinfectants, sweating etc. This is the most frequent reaction, is limited to the contact area and is characterised by itching, irritation and blistering at the side of contact.

## Appendix III

### Mast cell tryptase

1. Mast cells are found within many tissues including lung, intestine and skin. Tryptase is a protease enzyme which acts via widespread protease activated receptors (PARs). There are two types of tryptase; α and β, each of which has several subtypes. Mature β-tryptase is stored in granules in mast cells and is released during anaphylaxis by a calcium-dependent mechanism. α-tryptase is secreted constitutively by mast cells and is the form normally found in the blood in health. α-tryptase is elevated in systemic mastocytosis. Pro-β-tryptase is also secreted constitutively by mast cells.
2. The normal functions of tryptase include modulation of intracellular calcium ion and intestinal chloride ion transport in addition to mediating relaxation of smooth muscle in vascular endothelium and bronchial smooth muscle. In inflammation, tryptase stimulates the release of pro-inflammatory interleukins and stimulates further tryptase secretion by neighbouring mast cells.
3. A variety of pathways may combine to produce anaphylaxis, depending on the trigger and the susceptibility of the patient. Typically anaphylaxis results from mast cell activation, and typically this will cause release of mast cell β-tryptase into the circulation.
4. Anaphylaxis may be caused by basophil or complement activation, both of which are not associated with a rise in mast cell tryptase. A raised tryptase concentration is therefore a marker of anaphylaxis, but anaphylaxis does not always cause a raised concentration. The greater the rise in concentration, the greater the probability this was caused by an anaphylactic reaction but a normal concentration does not disprove anaphylaxis.
5. A rise in serum tryptase simply indicates mast cell degranulation and does not discriminate between allergic and non-allergic anaphylaxis. It has been stated that the rise in serum tryptase concentration may be less marked in non-allergic anaphylaxis.
6. During anaphylaxis, tryptase peaks by approximately 1 h and its half-life in the circulation is about 2 h: the concentration therefore falls rapidly following an anaphylactic reaction.
7. The most widely used assay for tryptase is the ImmunoCAP® fluorescence enzyme immunoassay (FEIA) which measures the sum of α and β-tryptase concentrations. A raised resting concentration is usually due to α-tryptase, secreted constitutively by the increased numbers of mast cells in systemic mastocytosis.
8. Although the current tryptase assay has a high specificity, its sensitivity is relatively low and some cases of anaphylaxis may be missed.
9. The sensitivity and specificity of the tryptase assay depends on the cut-off point that is chosen to indicate anaphylaxis. Because there is a wide variation in the base-line plasma concentration of tryptase, the change in tryptase concentration is more helpful than the absolute concentration in reaching a diagnosis.
10. Intravenous fluid replacement is usually required during treatment of a reaction. This fluid will dilute the blood and causes a fall in the concentration of tryptase measured. This must be taken into account when evaluating the measured concentration.
11. Following death, the circulation stops and tryptase is no longer removed from the circulation; samples taken postmortem are helpful in evaluating whether the terminal events were related to anaphylaxis. A raised tryptase concentration found at autopsy has lower predictive value than a sample taken during life. β-tryptase may be raised in trauma or myocardial infarction.

## Appendix IV

### Useful websites

http://www.aagbi.org

The Association of Anaesthetists of Great Britain and Ireland

http://www.bsaci.org

The British Society for Allergy and Clinical Immunology

http://www.immunology.org

The British Society for Immunology

http://www.resus.org.uk

Resuscitation Council UK

http://www.eaaci.net

The European Academy of Allergology and Clinical Immunology

http://www.mhra.gov.uk

The Medicines and Healthcare products Regulatory Agency
