# Supplementary material for: Suspected Anaphylactic Reactions Associated with Anaesthesia
Source: Anaesthesia. 2009 Feb;64(2):199–211. doi: 10.1111/j.1365-2044.2008.05733.x (PMC3082210; doi:10.1111/j.1365-2044.2008.05733.x)
Supplement: Supplementary file 1 [file ana0064-0199-SD1.doc]

**APPENDIX S1**

**Anaesthetic Anaphylaxis Referral Form**

**Patient details**

Name……………………………………………………………....................................

Date of birth …./…./…….. Hospital / NHS Number ………………………….

Address ………….………………………………………………………...................

……………………………………………………… Telephone …………………

**Referring clinician (address for correspondence)**

Name…………………………………………………………………...

Address…………..………….…………………………………………

………………………………………………………………………….

Telephone…………………… Fax: ……………………. Email ……………………

**Anaesthetist (if different from above)**

Name…………………………………………………………………...

Address…………..………….…………………………………………

………………………………………………………………………….

Telephone…………………… Fax: ……………………. Email ……………………

**Patient’s GP**

Name…………………………………………………………………...

Address…………..………….…………………………………………

………………………………………………………………………….

Telephone…………………… Fax: ……………………. Email ……………………

**Surgeon**

Name…………………………………………………………………...

Address…………..………….…………………………………………

………………………………………………………………………….

Telephone…………………… Fax: ……………………. Email ……………………

**Date of the reaction …./…./…….. Time of onset of reaction …./….h (24h clock)**

**Suspected cause of the reaction**

1) ……………………….. 2) …………………..…… 3) ……………………..…

**Proposed surgical procedure:……………………………………..**

Was surgery completed? Yes □ No □

If ‘no’, has another date for surgery being scheduled? Yes □ No □

Urgency of future surgery.……………………………………………………………...

**Details of the reaction**

| Symptom/ Sign | Onset Time  (24 h clock) | Time resolved (24 h clock) | Severity (Mild/Moderate/Severe) |
| --- | --- | --- | --- |
| Hypotension |  |  | Lowest BP / mmHg |
| Tachycardia |  |  |  |
| Bradycardia |  |  |  |
| Bronchospasm |  |  |  |
| Cyanosis/ desaturation |  |  | Lowest SpO2 |
| Angioedema |  |  |  |
| Urticaria |  |  |  |
| Arrhythmia |  |  |  |
| Flushing |  |  |  |
| Itching |  |  |  |
| Other (specify) |  |  |  |

**Drugs administered *BEFORE the onset of the reaction*. In addition, please include time of tracheal intubation, LMA insertion, and any other relevant event**

| Drug/Procedure | Time over which administered  (‘STAT’ or in min:sec) | Time (24 hr clock) | Route |
| --- | --- | --- | --- |
|  |  |  |  |
|  |  |  |  |
|  |  |  |  |
|  |  |  |  |
|  |  |  |  |
|  |  |  |  |
|  |  |  |  |
|  |  |  |  |
|  |  |  |  |
|  |  |  |  |
|  |  |  |  |

**Intravenous fluids given *BEFORE the onset of the reaction* with approximate start times**

1. ………………….. _____:_____
2. ..………………… _____:_____
3. ………………….. _____:_____

**Drugs given *AFTER*  *the onset of the reaction***

| Drug / Fluid | Time over which administered  (‘STAT’ or in min:sec) | Time (24 hr clock) | Route |
| --- | --- | --- | --- |
|  |  |  |  |
|  |  |  |  |
|  |  |  |  |
|  |  |  |  |
|  |  |  |  |
|  |  |  |  |
|  |  |  |  |
|  |  |  |  |
|  |  |  |  |
|  |  |  |  |
|  |  |  |  |
|  |  |  |  |
|  |  |  |  |

**Intravenous fluids given *AFTER the onset of the reaction* with approximate start times**

1. ………………….. _____:_____
2. ..………………… _____:_____
3. ………………….. _____:_____
4. ………………….. _____:_____

**Comments on response to treatment** …………………………………………………………………………………………..…………………………………………………………………………………………..

**Outcome**

Survived: Yes □ No □

Transfer to:

Ward □ HDU □ ICU □ Other …………………………………

**Anaesthetic techniques and procedures**

Latex free environment? Yes □ No □

Central venous access

Time: ……h Skin prep ……………………… Type of CVC ……………………

Was a coated catheter used? Yes □ No □

Neuraxial blockade

Spinal □ Epidural □ Epi-spinal □ Skin Prep……………………………..

| Drug/Procedure | Time over which administered  (‘STAT’ or in min:sec) | Time (24 hr clock) | Route |
| --- | --- | --- | --- |
|  |  |  |  |
|  |  |  |  |
|  |  |  |  |

Peripheral nerve blockade

Type of block(s) :………………………… Skin Prep ……………………

Drugs given for peripheral nerve blockade.

| Drug | Time over which administered  (‘STAT’ or in min:sec) | Time (24 hr clock) | Route |
| --- | --- | --- | --- |
|  |  |  |  |
|  |  |  |  |
|  |  |  |  |

Urethral catheterisation

Time ………h Antiseptic solution …………………………………. …...

Urethral lubrication/local anaesthetic.………………………………………….

Type of catheter (eg latex, silastic etc)…………………………………………

**Skin preparation for surgery and start of surgery**

Time skin preparation ………………h Skin Prep ………………………………….

Time surgery commenced: ………....h

Time surgery completed …………… h

**Investigations performed prior to referral (please give results if known)**

Were blood samples taken for Mast Cell Tryptase measurement? Yes □ No □

First sample Time___:___ Date___/___/____ Result………….

Second sample Time___:___ Date___/___/____ Result……….....

Third sample Time___:___ Date___/___/____ Result………….

Other bloods tests:

Test:…………………Time___:___ Date___/___/____ Result………………………

Test:…………………Time___:___ Date___/___/____ Result………………………

N.B. It is the anaesthetist’s responsibility to obtain the results from the laboratory

Case discussed at a multidisciplinary meeting? Yes □ No □

Reported to the MCA? Date___/___/____ By whom? ……………………………………

Reported to the AAGBI National Anaphylaxis database? Date___/___/____

Please send the completed form to the specialist investigation clinic together with:

- Photocopy of the anaesthetic record and any previous anaesthetic records
- Photocopy of the prescription record
- Photocopy of the recovery-room documentation
- Photocopy of any relevant ward documentation

*Please file a copy of this form in the patient’s casenotes and keep a copy for your own records.*

**APPENDIX V**

Useful websites

http://[www.aagbi.org](http://www.aagbi.org/)

The Association of Anaesthetists of Great Britain and Ireland

http://[www.bsaci.org](http://www.bsaci.org/)

The British Society for Allergy and Clinical Immunology

http://[www.immunology.org](http://www.immunology.org/)

The British Society for Immunology

http://[www.resus.org.uk](http://www.resus.org.uk/)

Resuscitation Council UK

http://[www.eaaci.net](http://www.eaaci.net/)

The European Academy of Allergology and Clinical Immunology

http://[www.mhra.gov.uk](http://www.mhra.gov.uk/)

The Medicines and Healthcare products Regulatory Agency
